# Genetic characterisation of antimicrobial resistance and virulence genes in *Staphylococcus aureus* isolated from commercial broiler chickens in the Durban metropolitan area, South Africa

**DOI:** 10.4102/jsava.v88i0.1416

**Published:** 2017-05-04

**Authors:** Nelisiwe Mkize, Oliver T. Zishiri, Samson Mukaratirwa

**Affiliations:** 1 School of Life Sciences, University of KwaZulu-Natal, South Africa

## Abstract

Antimicrobial resistance of *Staphylococcus aureus* in human and veterinary medicine is a serious worldwide problem. The aim of this study was to investigate the prevalence of *S. aureus* in commercial broiler chickens as well as to establish antimicrobial susceptibility and the distribution of genetic determinants conferring resistance and virulence. One hundred and ninety-four samples were aseptically collected from broiler chicken slaughterhouses and retail outlets around the Durban metropolitan area in South Africa. Microbiological and molecular methods were used to detect the presence of *S. aureus* as well as its resistance- and virulence-associated genes. Polymerase chain reaction (PCR) was used to confirm the presence of *S. aureus* by amplifying the *nuc* gene. Approximately 54% of 194 samples were positive for *S. aureus*. The disc diffusion technique was used to investigate antimicrobial susceptibility profiles of the *S. aureus* isolates to a battery of 10 antimicrobial agents, namely ampicillin, chloramphenicol, gentamicin, erythromycin, cefoxitin, kanamycin, streptomycin, tetracycline, vancomycin and trimethoprim. The results demonstrated that *S. aureus* isolates of abattoir origin had a high level (79.4%) of resistance to tetracycline, followed by ampicillin, vancomycin, cefoxitin, trimethoprim, erythromycin and streptomycin with resistance rates of 65.1%, 61.9%, 60.3%, 58.7%, 57.1% and 46.0%, respectively. *Staphylococcus aureus* isolates of retail origin exhibited higher antimicrobial resistance prevalence rates than those of abattoir origin. Tetracycline had the highest resistance rate (100%), followed by cefoxitin (91.7%), erythromycin (83.3%), streptomycin (83.3%) and kanamycin (66.7%). All isolates were resistant to two or more antimicrobial agents. Out of the four virulence genes that were screened, only two were detected (coagulase and protein A); however, their prevalence rates were very low. All antimicrobial resistance genes screened were detected (*mecA, BlaZ* and *tetK*), although their prevalence did not correspond with antimicrobial susceptibility testing.

## Introduction

*Staphylococcus aureus* is an opportunistic bacterium that is part of the normal commensal flora in humans and livestock. It is considered to be the most pathogenic species of the genus *Staphylococcus* (Quinn & Markey 2003) and to be a significant cause for a wide range of avian diseases including arthritis, staphylococcal septicaemia, synovitis, omphalitis and infection of the yolk sac (Mead & Dodd 1990; Smyth & McNamee 2001). These staphylococcal infectious diseases of chickens are an economic threat and are regarded as a global burden (Lowder et al. 2009). The presence of, especially, asymptomatic *S. aureus* infections has the potential to contaminate chicken carcasses (Köck et al. 2013; Olivier et al. 1996). This implies an increased risk for contaminated chicken meat and its products to be transported to retail outlets and subsequently to the consumer. The presence of *S. aureus* in contaminated chicken meat products may result in the production of thermostable enterotoxins that may cause staphylococcal food poisoning in humans (Balaban & Rasooly 2000). This results in a wide range of infections such as gastroenteritis, heat shock-like syndrome, skin infections, respiratory infections, urinary tract infections and immune-mediated diseases (Balaban & Rasooly 2000; Larsen, Sloth & Elsberg 2000).

*Staphylococcus aureus* virulence is complex and depends on an array of virulence genes. Virulence genes involved in this microorganism are clustered under two categories, namely genes coding for cell-surface-associated (adherence) and secreted (exotoxins) factors (Diep & Otto 2008). *Staphylococcus aureus* achieves colonisation of the host through production of various exoproteins (Salasia et al. 2004). A typical example of a well-known exoprotein is protein A, which is considered to be an important virulence factor (Agius et al. 2007). Furthermore, the *spa* gene, which is mostly used for typing of *S. aureus*, encodes for protein A. The coagulase (*coa*) gene is another example of a *S. aureus* virulence gene that is regarded as important, because it plays an essential role in the alliance with other genes to survive inside host cells and to invade immune system cells in the host. Most virulence genes in *S. aureus* are known to be associated with staphylococcal food poisoning (Balaban & Rasooly 2000).

Livestock-associated antimicrobial resistance of *S. aureus* to several antimicrobial agents has been reported (Aarestrup 1999; Ateba et al. 2010; Hanson et al. 2011). There is a potential for such antimicrobial resistance to be passed on from food-producing animals to humans through food and direct contact with contaminated carcasses. *Staphylococcus aureus* has a potential to be resistant to any group of antimicrobial agents, and its resistance has been reported in veterinary and human health sectors. Among all types of resistant *S. aureus*, methicillin-resistant *S. aureus* (MRSA) is regarded as one of the most crucial, because it was proven to be the cause of acquired infections associated with a high rate of bacterial mortality worldwide (Tiemersma et al. 2004). There are numerous reports in which MRSA strains have been isolated in livestock meat (beef, pork and chicken) and in different food products such as diary milk and its products. The presence of MRSA in food products poses a threat that it can potentially lead to the spread of MRSA to consumers through the food chain (De Neeling et al. 2007; Voss et al. 2005; Wulf & Voss 2008). Antimicrobial resistance genes can be transmitted across species using mobile genetic elements (MGEs), which are DNA (deoxyribonucleic acid) fragments that carry both virulence and resistant determinants. Furthermore, they produce enzymes that allow them to be transferred and integrated into a new host’s DNA. The transfer of MGEs among cells is known as horizontal gene transfer (HGT), and it can occur among prokaryotes and eukaryotes (Malachowa & DeLeo 2010). This mechanism plays a vital role in bacterial evolution. There are many different types of MGEs, such as gene cassettes, insertion sequences, bacteriophages, transposons and plasmids (Malachowa & DeLeo 2010; Mascaretti 2003).

Contamination of chicken meat by *S. aureus* has been detected in numerous countries including the Netherlands, Japan, Brazil, USA and Nigeria (De Boer et al. 2009; Islam et al. 2014; Kitai et al. 2005; Kwon et al. 2006; Momtaz et al. 2013; Ugwu et al. 2015). In South Africa, there is a paucity of information on MRSA isolated from chicken meat. Furthermore, there is a literature dearth on the prevalence and genetic characterisation of *S. aureus* in chicken meat and its products. Against this background, this study aimed to investigate the prevalence of *S. aureus* in chicken samples and to further analyse the detected isolates by screening for genetic determinants carried by this bacterium encoding for virulence and antimicrobial resistance.

## Materials and methods

### Sample collection

Broiler chicken samples (caecum, faeces and retail meat) were collected from poultry slaughterhouses and retail outlets within the Durban metropolitan area in the KwaZulu-Natal Province of South Africa as described by Zishiri, Mkhize and Mukaratirwa (2016). In summary, abattoir samples were collected on the days of slaughter between March and October 2014, in batches of 25 per month. A total of 200 samples were randomly collected over the 8-month period; however, 114 samples were randomly selected for this study. A total of 30 samples were randomly purchased from 10 retail outlets (three samples per outlet) around the Durban metropolitan area between May and November 2015. Moreover, 50 chicken faecal samples were randomly collected at markets around the Durban metropolitan area during the same period. The markets were defined as open places where informal entrepreneurs commercially sell live broilers emanating from different areas in KwaZulu-Natal. A total of 194 samples were thus examined in this study. All samples were aseptically collected in plastic screw-top tubes containing 45 mL of 0.1% w/v peptone-water and stored on ice until transported to the University of KwaZulu-Natal, Westville Campus in Durban for analysis.

### Detection of *Staphylococcus aureus*

Firstly, enrichment was conducted by taking 10 mL of rinse peptone-water from the collected samples into clean sterile test tubes and incubated at 37 °C for 24 h. After incubation, 0.1 mL aliquots from the peptone-water samples were inoculated into the tubes containing 10 mL of brain–heart infusion (BHI) broth and incubated at 37 °C for 24 h. After enrichment, a loopful of the broth culture was streaked onto plates containing mannitol salt agar and incubated at 37 °C for 24 h. Typical phenotypic characteristics of yellow colonies with yellow zones were regarded as positive for *S. aureus.* Suspected *S. aureus* colonies were selected and inoculated on BHI broth and incubated while shaking at 37 °C for 24 h. The resulting culture was used for DNA extraction and some was used for antimicrobial susceptibility tests. The remaining culture was used for 60% glycerol stocks that was then stored at -80 °C.

### DNA extraction

Genomic DNA of all *S. aureus* isolates was extracted from the culture using the Zymo Research Fungal and Bacterial Genomic DNA MiniPrep^TM^ kit following the manufacturer’s instructions. A positive *S. aureus* control was prepared by isolating genomic DNA from a reference strain of known *S. aureus* broth culture. After DNA extraction, a NanoDrop spectrophotometer was used to check the concentration and quality of the isolated DNA. The extracted DNA was stored at -20 °C until used for molecular confirmation of the species and screening for virulence and antimicrobial resistance genes.

### Molecular confirmation of *Staphylococcus aureus*

Polymerase chain reaction (PCR) was used to amplify the *nuc* gene for the confirmation of detected *S. aureus* in the isolates. The *nuc* gene primers that were used had been previously described by Brakstad, Aasbakk and Maeland (1992) (Table 1). PCR was carried out in a total volume of 25 µL containing 12.5 µL DreamTaq Green PCR Master Mix, 1 µL *nuc* primer (forward), 1 µL *nuc* primer (reverse), 4 µL template DNA and 6.5 µL dH_2_O. Amplification was carried out in a thermocycler using 34 cycles consisting of denaturation for 30 s at 95 °C, annealing for 30 s at 57 °C, extension for 1 min at 72 °C and final extension for 5 min at 72 °C. PCR products were run on a 1.5% agarose gel using electrophoresis, stained with gel red at 70 volts for 60 min and visualised under UV light using a gel documentation system (Bio ChemiDoc^TM^ MP imaging system).

**TABLE 1:** Sequences of oligonucleotide primers used to target genetic determinants responsible for species confirmation, virulence and resistance in *Staphylococcus aureus*.

Target gene	Primer sequence (5’ 3’)	Product size (bp)	References
*nuc*	F: GCGATTGATGGTGATACGGTT	270	Brakstad et al. (1992)
	R:AGCCAAGCCTTGACGAACTA AAGC		
*coa*	F: CGA GAC CAA GAT TCA ACA AG	730	Aslantas et al. (2007)
	R: AAA GAA AAC CAC TCA CAT CA		
*spa*	F: CAA GCA CCA AAA GAG GAA	320	Frenay et al. (1996)
	R: CAC CAG GTT TAA CGA CAT		
*sea*	F: GCA GGG AAC AGC TTT AGGC	521	Monday and Bohach (1999)
	R: GTT CTG TAG AAG TAT GAAACA CG		
*see*	F: TAC CAA TTA ACT TGT GGA TAG AC	171	Monday and Bohach (1999)
	R: CTC TTT GCA CCT TAC CGCA		
*mecA*	F: AAAATCGATGGTAAAGGTTGGC	532	Strommenger et al. (2003)
	R: AGTTCTGCAGTACCGGATTTGC		
*BlaZ*	F: ACTTCAACACCTGCTGCTTTC	240	Martineau et al. (2000)
	R: TAGGTTCAGATTGGCCCTTAG		
*tetK*	F: TTAGGTGAAGGGTTAGGTCC	718	Aarestrup et al. (2000)
	R: GCAAACTCATTCCAGAAGCA		

### Antimicrobial susceptibility testing

Antimicrobial resistance of the 104 *S. aureus-*positive isolates were tested against 10 antimicrobial agents using the Kirby-Bauer disc diffusion method on Mueller–Hinton agar following the guidelines of the Clinical and Laboratory Standards Institute ([CLSI] 2013). The 10 antimicrobial agents selected, based on their common use in the poultry industry and for the treatment of human infections, were ampicillin (25 µg), chloramphenicol (30 µg), erythromycin (30 µg), cefoxitin (30 µg), gentamicin (30 µg), kanamycin (30 µg), streptomycin (25 µg), tetracycline (30 µg), trimethoprim (5 µg) and vancomycin (30 µg). Multiple-drug resistance was assumed when an isolate was resistant to two or more antimicrobial agents. The Oxoid antibiotic discs with the desired concentration of each antibiotic as per the CLSI guidelines were used. Firstly, Mueller–Hinton agar was inoculated with 0.1 mL of nutrient broth samples, which had previously been inoculated with a loopful of glycerol stocks of positive samples and then incubated at 37 °C for 24 h. The culture was spread on the agar with a sterile swab for even distribution of *S. aureus*; thereafter, Oxoid antibiotic discs were evenly placed on plates and the plates were incubated at 37 °C for 24 h. The inhibition zones were measured and scored as sensitive (S), intermediate susceptibility (I) or resistant (R) according to the CLSI recommendations. *Staphylococcus aureus* ATCC 25923 was used as a reference strain for antibiotic disc control (Treangen et al. 2014).

### Screening for virulence and antimicrobial resistance genes

The broiler chicken and faecal isolates that tested positive for *S. aureus* were screened for seven genetic determinants. Among these, four encode for virulence (*spa, coa, sea* and *see*) and three encode for antimicrobial resistance (*mecA, BlaZ* and *tetK*). Screening of virulence and antimicrobial resistance genes was carried out using PCR with previously described oligonucleotide primers (Table 1). The reactions were performed in a final volume of 25 µL each made by 12.5 µL DreamTaq Green PCR Master Mix, 1 µL primer (forward), 1 µL primer (reverse), 4 µL of template DNA and 6.5 µL dH_2_O. PCR conditions described by the original designers of primers were used without any amendments because of their reported reliability (Table 1). Following completion of reactions, 7 µL of the PCR products was analysed by 1.5% gel electrophoresis technique using 1X TBE as a medium buffer. Pictures were then taken using UV light gel documentation (Bio ChemiDoc^TM^ MP imaging system).

## Results

### Species confirmation

**FIGURE 1:**
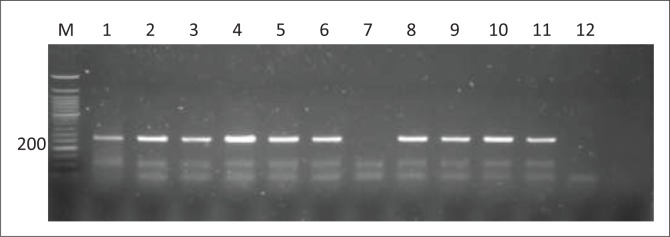
Agarose (1.5%) gel electrophoresis image of the *nuc* gene (270 bp). Lane M is a 50-bp DNA ladder, lanes 1 to 10 are test samples, lane 11 is a positive control and lane 12 is a negative control.

One hundred and four (53.6%) of the 194 broiler chicken and faecal samples tested positive for *S. aureus*. The *S. aureus*-positive isolates were obtained from samples from different origins, which included 32.5% caecum samples from the abattoirs, 6.2% different chicken organs from retail outlets and 15% faecal samples from the local markets around Durban. Figure 1 depicts a gel image with 270 bp PCR gene amplicons demonstrating the presence of the *nuc* gene that was amplified on representative *S. aureus*-positive isolates.

### Antimicrobial susceptibility profiles

Resistance to antimicrobial agents used is depicted in Table 2. When *S. aureus* isolates of abattoir origin were evaluated for antimicrobial susceptibility profiles, the highest level of resistance (79.4%) was against tetracycline, followed by ampicillin, vancomycin, cefoxitin, trimethoprim, erythromycin and streptomycin with resistance rates of 65.1%, 61.9%, 60.3%, 58.7%, 57.1% and 46.0%, respectively. The lowest level of resistance observed involved gentamicin, with only 15.9% of the isolates being resistant to this drug. *Staphylococcus aureus* isolates in samples of retail origin exhibited higher prevalence rates of antimicrobial resistance compared to those of abattoir origin. All retail isolates were resistant to tetracycline (100%). Similarly, high antimicrobial resistance rates were demonstrated against cefoxitin (91.7%), erythromycin (83.3%), streptomycin (83.3%) and kanamycin (66.7%). Low levels of resistance were observed for gentamicin (25%), and no isolates exhibited resistance to either ampicillin or vancomycin. Lastly, *S. aureus* isolates from faecal samples were highly resistant to kanamycin (79.3%), cefoxitin (76.0%), tetracycline (69.0%), erythromycin (62.1%), streptomycin (62.1%), trimethoprim (58.6%), chloramphenicol (69.0%) and gentamicin (55.2%). Low rates of resistance were observed against ampicillin (27.6%) and vancomycin (14.0%) in the chicken faecal isolates. All 104 *S. aureus* isolates tested were resistant to two or more antimicrobial agents used (Table 3).

**TABLE 2:** Prevalence rates for antimicrobial susceptibility tests on *Staphylococcus aureus* isolated from broiler chicken samples of different origins.

Antibiotics	Abattoir samples (*n* = 63)			Retail samples (*n* = 12): number of isolates (%)			Faecal samples (*n* = 29)		
		
	R	I	S	R	I	S	R	I	S
AMP	41 (65.1)	1 (1.6)	21 (33.3)	0 (0.0)	0 (0.0)	12 (100.0)	8 (27.6)	4 (14.0)	17 (58.6)
C	22 (34.9)	4 (6.4)	37 (58.7)	5 (41.7)	0 (0.0)	7 (58.3)	20 (69.0)	1 (3.4)	8 (27.6)
CN	10 (15.9)	2 (3.2)	51 (81.0)	3 (25.0)	1 (8.3)	8 (66.7)	16 (55.2)	4 (14.0)	9 (31.0)
E	36 (57.1)	15 (23.8)	12 (19.0)	10 (83.3)	2 (16.7)	0 (0.0)	18 (62.1)	2 (6.9)	9 (31.0)
FOX	38 (60.3)	2 (3.2)	23 (36.5)	11 (91.7)	0 (0.0)	1 (8.3)	22 (76.0)	1 (3.4)	6 (20.7)
K	21 (33.3)	0 (0.0)	42 (66.7)	8 (66.7)	0 (0.0)	4 (33.3)	23 (79.3)	2 (6.9)	4 (13.8)
S	29 (46.0)	2 (3.2)	32 (50.8)	10 (83.3)	0 (0.0)	2 (16.7)	18 (62.1)	4 (14.0)	7 (24.1)
TE	50 (79.4)	4 (6.4)	9 (14.3)	12 (100)	0 (0.0)	0 (0.0)	20 (69.0)	1 (3.4)	8 (27.6)
VA	39 (61.9)	4 (6.4)	20 (31.7)	0 (0.0)	0 (0.0)	12 (100.0)	4 (14.0)	3 (10.3)	22 (75.9)
W	37 (58.7)	3 (4.8)	23 (36.5)	9 (75.0)	0 (0.0)	3 (25.0)	17 (58.6)	0 (0.0)	12 (41.4)

AMP, ampicillin; C, chloramphenicol; CN, gentamicin; E, erythromycin; FOX, cefoxitin; K, kanamycin; S, streptomycin; TE, tetracycline; VA, vancomycin; W, trimethoprim; R, resistant; I, intermediate susceptibility; S, susceptible.

The *S. aureus* isolated from the abattoirs demonstrated moderate to high (54.0%) multiple-drug resistance to cefoxitin and ampicillin as well as moderate (47.6%) resistance to cefoxitin, tetracycline and vancomycin (Table 3). Low multiple-drug resistance ranging from 8.0% to 36.5% was evident in antibiotic combinations involving cefoxitin, trimethoprim, gentamicin, tetracycline, vancomycin, erythromycin and streptomycin. The *S. aureus* isolates from retail samples exhibited moderate to high multiple-drug resistance ranging from 50% to 75% to a combination of antimicrobials involving cefoxitin, kanamycin, trimethoprim, tetracycline, erythromycin and streptomycin as depicted in Table 3. Moderate (41.7%) multiple-drug resistance was exhibited in antibiotic combinations such as cefoxitin, trimethoprim, tetracycline, kanamycin, streptomycin, erythromycin and chloramphenicol. The *S. aureus* isolates that emanated from chicken faecal samples demonstrated moderate to high multiple-drug resistance that ranged from 51.7% to 58.6% to antibiotic combinations that included cefoxitin, streptomycin, tetracycline and kanamycin (Table 3) and moderate multiple-drug resistance ranging from 34.5% to 41.4% to antibiotic combinations that included cefoxitin, chloramphenicol, gentamicin, tetracycline, erythromycin, kanamycin, trimethoprim and streptomycin. Low multiple-drug resistance ranging from 10.3% to 24.1% was evident in antibiotic combinations that included cefoxitin, chloramphenicol, vancomycin, gentamicin, tetracycline, streptomycin, erythromycin and ampicillin.

**TABLE 3:** Multiple-drug resistance patterns of *Staphylococcus aureus* isolates.

Antimicrobial resistance patterns	Number of isolates (%)		

	Abattoir samples (*n* = 63)	Retail samples (*n* = 12)	Faecal samples (*n* = 29)
FOX, AMP	34 (54.0)	0 (0.0)	7 (24.1)
FOX, S, TE	23 (36.5)	9 (75.0)	15 (51.7)
FOX, AMP, K	12 (19.0)	0 (0.0)	7 (24.1)
FOX, C, CN	8 (12.7)	3 (25.0)	10 (34.5)
FOX, K, TE	16 (25.4)	7 (58.3)	17 (58.6)
FOX, W, S	20 (31.7)	7 (58.3)	12 (41.4)
FOX, TE, VA	30 (47.6)	0 (0.0)	4 (13.8)
FOX, CN, TE, K	8 (12.7)	3 (25.0)	11 (37.9)
FOX, S, W, E	16 (25.4)	6 (50.0)	11 (37.9)
FOX, E, S, VA	14 (22.2)	0 (0.0)	4 (13.8)
FOX, K, CN, TE	8 (12.7)	3 (25.0)	11 (37.9)
FOX, TE, E, C	16 (25.4)	5 (41.7)	12 (41.4)
FOX, W, TE, K, S	11 (17.5)	5 (41.7)	11 (37.9)
FOX, K, W, TE, E	12 (19.0)	6 (50.0)	11 (37.9)
FOX, AMP, S, K, C	11 (17.5)	0 (0.0)	7 (24.1)
FOX, C, VA, CN, TE	6 (9.5)	0 (0.0)	3 (10.3)
FOX, TE, K, E, C	12 (19.0)	4 (33.3)	12 (41.4)
FOX, W, CN,TE, VA, E	5 (7.9)	0 (0.0)	4 (13.8)
FOX, VA, E, C,TE, W	12 (19.0)	0 (0.0)	4 (13.8)
FOX, E, S, VA, TE, K	9 (14.3)	0 (0.0)	4 (13.8)

AMP, ampicillin; C, chloramphenicol; CN, gentamicin; E, erythromycin; FOX, cefoxitin; K, kanamycin; S, streptomycin; TE, tetracycline; VA, vancomycin; W, trimethoprim.

### Prevalence of virulence and antimicrobial resistance genes in *****Staphylococcus aureus***** in broiler chickens and faecal samples

**FIGURE 2:**
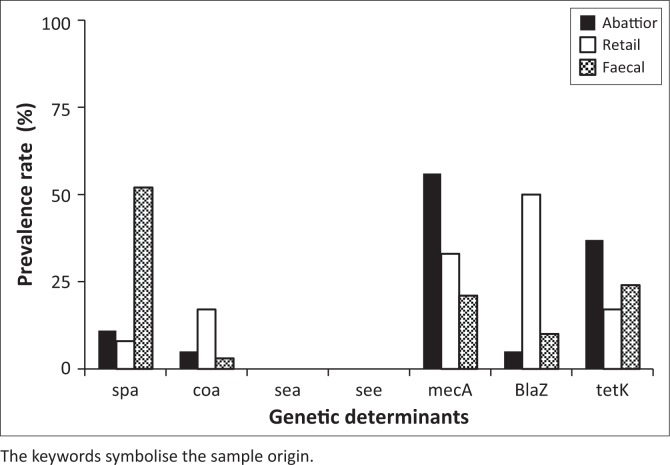
Prevalence rates of genetic determinants encoding for virulence and resistance in *Staphylococcus aureus* isolates.

Antimicrobial resistance genes were detected in all 104 *S. aureus* isolates, regardless of antimicrobial susceptibility phenotypes. The prevalence rates of the genes are depicted in Figure 2. Of the four virulence genes screened from all the isolates, only two (*spa* and *coa*) were detected. In isolates from the abattoirs, retail and faecal samples, the prevalence rates observed for the *spa* gene were 11%, 8% and 52%, respectively. However, the prevalence rate of the *coa* virulence gene in abattoir, retail and chicken faecal samples was low in the magnitude of 5%, 17% and 3%, respectively. All three antimicrobial resistance genes screened from 104 *S. aureus* isolates were detected (Figure 2). Prevalence rates for the gene encoding for methicillin resistance (*mecA*) were 56%, 33% and 21% from isolates from abattoir, retail and faecal samples, respectively. The beta lactamase gene (*BlaZ*) was detected in 4.8% of isolates of abattoir origin, 50% of isolates of retail origin and 10.3% of isolates from faecal samples. Lastly, the prevalence rates for the *tetK* gene encoding for tetracycline resistance were 37%, 17% and 24% for abattoir, retail and faecal samples, respectively, as depicted in Figure 2.

## Ethical considerations

Animal studies have been approved by the appropriate ethics committee of the University of KwaZulu-Natal (Reference: 012/15/Animal); therefore, they have been performed in accordance with the ethical standards laid down in the 1964 Declaration of Helsinki and its later amendments.

## Discussion

The increasing reports of antimicrobial resistance of *S. aureus* associated with food-producing animals such as poultry have been a driving force for surveillance studies focusing on detection and assessment of antimicrobial resistance profiles (Gundogan et al. 2005; Nemati et al. 2008). Most reported that there is a continuous increase in the incidence of antimicrobial resistance in *S. aureus*. Most researchers have speculated that this increase is a result of unwarranted and injudicious use of antimicrobial agents in veterinary and human medicine (Aarestrup 1999; Teuber 2001), creating selection pressure that can be disseminated to the environment through various factors (such as direct contact and via the food chain), with the potential to pose threats to animal and human health.

This study demonstrated that out of 194 broiler chicken samples tested, only 104 (53.6%) samples were confirmed positive for *S. aureus* based on the presence of the *nuc* gene amplicon. The results are almost in agreement with findings from similar studies by Kitai et al. (2005) and Shareef, Farag and Al-Ruthwani (2012), which yielded prevalence rates of 44% and 47%, respectively. However, the reports from the previous authors were also relatively high compared to findings from similar studies by Hanson et al. (2011) and Momtaz et al. (2013), with contamination prevalence rates of 17.80% and 28.05%, respectively. Furthermore, these findings are relatively low compared to reports by Islam et al. (2014), where 95% of chicken samples used for the study were contaminated by *S. aureus.*

The presence of *S. aureus* in chicken meat at an abattoir presents a possibility for this pathogen to being disseminated into the community through slaughterhouse workers during meat handling as well as during unintentional transportation of contaminated meat to retail outlets. Moreover, in this study, *S. aureus* was detected from chicken samples collected at retail level and from faecal samples collected from local open markets where there are traders and clients coming into close and direct contact on a daily basis. This implies that there is a potential for consumers to acquire the pathogen through ingestion of contaminated broiler chicken meat from retail trade and also through exposure to the contaminated environment as faecal samples were found contaminated with *S. aureus*. Faeces are also regarded as a major vehicle for the dissemination of pathogens from avian species.

Among the four virulence genes screened, only the *spa* and *coa* genes were detected. The prevalence rates of these two genes observed were higher compared to findings from a similar study by Bunnoeng et al. (2014), where 0.0% and 2.5% were observed for the *coa* and *spa* genes, respectively. However, the findings for the *sea* gene from the Bunnoeng et al. (2014) study are in concordance with our study where no enterotoxin genes were observed. The polymorphic *coa* and *spa* genes can be used to investigate the diversity of *S. aureus* (Vintov et al. 2003). The *coa* gene is a virulence gene that is also used to determine the coagulase status of *S. aureus* isolates. Relatively low prevalence rates of *coa* gene were detected in *S. aureus* isolates, and it can be concluded that the isolates were probably mostly coagulase negative. Coagulase negative *S. aureus* is non-pathogenic, but it does harbour some virulence genes at a low rate. Therefore, this information can be used as an explanation for the low prevalence rate of virulence genes obtained in this study because most of the isolates lacked the *coa* gene.

The availability and easy accessibility of antimicrobial agents have been a catalyst for their extensive use in the poultry industry to promote growth and to treat infections caused by various bacterial pathogens. Aarestrup (2005) reported that extensive use of antimicrobial agents both in small and in large quantities represents a health risk because it creates selection pressure for antimicrobial resistance. It is therefore crucial to monitor antimicrobial resistance profiles of bacteria isolated from livestock and humans, so that the information can be used to guide public health officials to encourage prudent use of antimicrobial agents in human and veterinary medicine (Cummings et al. 2013).

In this study, *S. aureus* isolates were highly resistant to tetracycline, ampicillin, cefoxitin, trimethoprim and erythromycin, but mostly susceptible to gentamicin and chloramphenicol (Table 2), and multiple-drug resistance was also observed for almost all the isolates (Table 3). These results correspond with the findings by Momtaz et al. (2013), Islam et al. (2014) and Ugwu et al. (2015). In all previous comparative studies, tetracycline resistance was the most prevalent compared to resistance to other antimicrobial agents. This confirms information provided by Huys et al. (2005) regarding tetracycline resistance as one of the most frequently occurring resistance phenotypes in *S. aureus* isolated from farming, processing and storage environments of poultry. Tetracycline is widely used in poultry industries worldwide, because it is relatively cheap and it has fewer side effects (Chopra & Roberts 2001). Extensive use of tetracyclines might be the reason behind high prevalence rates of resistant *S. aureus* isolates associated with chicken samples. In this study, the *tetK* gene encoding for tetracycline resistance was screened for all the samples, and the prevalence rates (Figure 2) of *S. aureus* isolates harbouring this gene were very low compared to the rate of isolates that exhibited tetracycline resistance during antimicrobial susceptibility testing. Because there is a pool of genes encoding for tetracycline resistance, genes that were responsible for the resistance in *S. aureus* were likely not part of this study.

Methicillin-resistant *S. aureus* is currently a major burden in veterinary and human medicine. This type of resistance is considered to be one of the most important and has been implicated in many animal and human illnesses that have resulted in high mortality. In this study, MRSA was detected in abattoir samples (56%), retail samples (33%) and faecal samples (21%) based on the presence of the *mecA* gene amplicon (Figure 2). Furthermore, results from antimicrobial susceptibility testing indicated that abattoir, retail and chicken faecal samples were highly resistant to the antibiotic cefoxitin (Table 2) in the magnitude of 60.3%, 91.7% and 76.0%, respectively. The *mecA* gene is regarded as a major gene encoding for MRSA, but there are other genes, namely *pbpB* (Pinho, De Lencastre & Tomasz 2001) and *murF* (Sobral et al. 2003) that have been demonstrated to play a role in MRSA. Febler et al. (2011) and Wulf and Voss (2008) reported that isolation of MRSA from livestock stimulated great interest in many researchers because of the impact it has in the food chain. Febler et al. (2011) further reported that consequences of livestock-associated *S. aureus* are often fatal, because they create treatment complications that are normally accompanied by multi-drug resistance. Therefore, it is crucial to monitor and mitigate the presence of MRSA in livestock especially poultry, because it is the most consumed source of animal protein globally with a potential to escalate the spread of MRSA in human beings.

In South Africa, research similar to this study is more focused on milk from dairy cows (Akindolire, Babalola & Ateba 2015; Ateba et al. 2010) than on chicken meat, simply because *S. aureus* is known to cause mastitis in cattle and it is a huge challenge to the dairy industry. Most importantly, it is considered to be one of the major sources of staphylococcal infections in humans. A balanced focus on investigations based on the presence of *S. aureus* and its resistance phenotypes is important for all livestock species.

## Conclusion

*Staphylococcus aureus* was detected in chicken and faecal samples collected from different areas within the Durban metropolitan area in South Africa. It is important to emphasise enforcement of hygienic meat production as well as the prudent use of antibiotics in order to mitigate the spread of multiple-drug resistant strains of *S. aureus*. The detection of drug-resistant strains such as MRSA should be intensified and measures should be taken to prevent the evolution of other strains of *S. aureus* that are resistant to other antibiotics. This study also demonstrated resistance to vancomycin, which is an important antibiotic for the treatment of resistant Gram-positive infections in humans.
